# Antitoxin in the Treatment of Diphtheria1Read before the Detroit Academy of Medicine, Detroit, Mich., March 11, 1895.

**Published:** 1895-06

**Authors:** Charles D. Aaron

**Affiliations:** 535 Woodward Avenue; Detroit, Mich.


### ANTITOXIN IN THE TREATMENT OF DIPHTHERIA.[1]

By CHARLES D. AARON, M. D., Detroit, Mich.

In treating of the subject of antitoxin, we shall divide it into three parts. We shall try to give an account of the new etiology of infectious and contagious diseases. We shall give a short synopsis of the steps taken by contemporary scientists, which led to the new therapeutics and, finally, we shall treat, in the light of both, the special subject of diphtheria.

First, then, as to the cause of these diseases, as they are interpreted by bacteriologists. Behring's law is now a part of the history of medicine and is fast becoming a rule in medical practice. It says[2] that the blood, and blood serum, of an individual which has been artificially rendered immune against a certain infectious disease, may be transferred into another individual with the effect to render the latter also immune, no matter how susceptible this animal is to the disease in question. This is not merely Jenner's inoculatory theory in detail, nor is it an amplification of it. Behring's law is fundamental and we shall have to rewrite our pathology and therapeutics in accordance with it. Jenner was a sort of genius, who anticipated these grand discoveries, such as bacilli pathogenesis is; but, as all geniuses, he gave not more than undefined, grand guesses. It was left to the splendid talents and to the patient industry of Pasteur, Behring, Roux, Kitasato,

Ehrlich and others to bring into the medical world the farthest reaching revolution it has ever experienced.

In order to put this important discovery clearly, let me state the following medical axiom—namely, that immunity against disease is always a chemical condition. It has been found that certain individuals are naturally not susceptible to certain diseases; but it has also been found that nothing could be attained by the transfusion of either their blood or of their blood serum; that on the other hand, however, the blood and the blood serum of such as had been affected by the pathological condition of any of the infectious or contagious diseases, acquired a certain reagent power; that, in short, an organic chemism took place.

It was also found that the lymph and the blood of a healthy organism do not constitute a habitat in which microbes can live; that, in fact, bacteria perish in them, probably through want of food; that, in short, healthy blood and lymph have bactericide power. The experiments of Behring, Büchner and others showed, further, that this insusceptibility of certain individuals and the bactericide power of good blood were due to albumenoids in dissolution in the serum. And they ascertained the fact, within which all the rest of antitoxin therapeutics is latent, that the sera of different animals have different degrees of bactericide effect. Again, another fine series of experiments proved that species which have natural immunity against certain diseases could furnish serum which, if inoculated into individuals of another, but susceptible species, had neutralizing power, provided, of course, the transfusion or rather intoxication proceeded in a graduated manner.

The next problem, however, which confronted these painstaking students, was to define this bactericide quality of transfused serum. The theory current up to about 1889 supposed that patients who had recovered from infectious or contagious diseases were capable of resisting another attack of the disease, simply because the first visit of microbes had left nothing for the newcomers to feed on, these having themselves already consumed all available cellular food. It was supposed, also, that the first had left behind them a kind of bacterial product, which was noxious to the second swarm of microbes, and that, consequently, the individual was practically immunized by the disease germs. This was the first step in the direction of Behring's great presentation and law. In 1890, Behring gave to the world the result of his investigation on artificial immunization. He called attention to several phenomena, which even bacteriologists had failed to notice. He had discovered that the bacilli produced a toxin, that this toxin was present in the blood serum of an infected animal, that the gradual intoxication of an animal rendered it immune against tetanus, and that this immunity was caused by the neutralizing effect which the blood serum of an infected animal has upon the toxins produced by tetanus bacilli. He further found that the toxin could be destroyed by the blood of an immunized animal, that this blood or blood serum of an infected animal would neutralize the toxin, even then, when it was transfused into other animals; and, lastly, that artificial immunity could be accomplished even by chemical poisons; that, in fact, the entire matter of immunization was a chemical process.

This was noticeable most interestingly in the not infrequent instances of spontaneous recovery from infectious diseases. A logical explanation of this phenomenon consists in the fact that in such cases the effect of the toxins, caused by the disease, is neutralized by the coincident presence of certain antitoxic constituents of the blood. These patients have immunity against such respective disease by the continued presence in the organism of residual antitoxin. The blood serum of animals which had been inoculated was now a workable quantity. Animals which had been inoculated with disease were saved by the administration of blood serum of immunized animals, in doses proportionate to the degree of antecedent intoxication. So the new etiology of germs was brought to bear upon a new therapeutics.

The man who now came to the aid of Behring was Prof. Ehrlich. Continuing the line of investigations which looked upon the subject of bacterial transfusion as a matter of chemistry rather than of microscopic biology, he brought to light the fact that not only did the blood possess bactericidal power, but also that along with it the blood contained the neutralizing reagents against the bacilli, an accompanying element which was, in fact, the seat of immune action. This we may now call by the general term of antitoxin. Along with Behring, it was shown by him and others that the injection of blood taken from immune animals rendered others immune, and that this impartition of immunity was entirely due to an element in the blood of the infected animal, which before had not been separated from the toxin, but which now was an attendant on it. So the new therapeutics began.

The problem resolved itself into the question how to ascertain the degree of immunity of the animal which had been made insusceptible by the administration of antitoxin and the degree of neutralizing capacity which its blood would have upon a known degree of intoxication. This antitoxin differs from the original toxalbumin in color, the first being whitish, the second yellowish ; in form, the first being crystalline, the second amorphous. They are differentiated, however, essentially by the fact that, whereas the toxalbumin is virulent in transfusion, the other is harmless. This last was no small item in the construction of the new therapeutical significance which antitoxin was bound to acquire.

Though we have arrived now at the subject of diphtheria, to which the presentation till now was preparatory, we must still attend to one item. We must explain the terms, natural immunity and artificial immunity. Natural immunity can be described somewhat like this : the white corpuscles of the blood produce a certain germicide property. This property scientific experiments have shown to reside in the nuclei of the proteid substance—let me here mention the nuclein of Dr. Vaughan—and it has been observed that there is an abnormal increase of leucocytes in those places which are infected by the disease. It is now established that the alexins are to be credited with this defensive capacity.

In regard to artificial immunity, we must not forget that there is a considerable difference between the alexins present in the leucocytes and the antitoxin, which is introduced into the organism. The difference consists in the difference of their respective methods of attack. The alexins destroy the germs directly by drawing them within the nuclei of the proteid substance and digesting them. The antitoxin can only act upon the corresponding poisons, if these have been generated by metabolism or have been introduced into the organism by means of intoxication. Again, the difference is one of biology as well. Alexins are animal and the antitoxin element is bacterial. Besides, the alexins are present in the blood alone, while the antitoxin agents pervade the tissues. Lastly, we may say that the striking fact of their difference is that, while alexins, though they give natural immunity to personal organisms, cannot impart it by transfusion ; specific immunity, however, can be established in a foreign organism by inoculation of antitoxin by means of serum.

It is unnecessary to state that we know of diphtheria bacilli since 1884, through the discoveries of Klebs and Löffler. Also that there are certain other bacilli, which resemble the original Klebs and Löffler bacilli very much; so that they may be regarded as two varieties of one species. We may, however, state that the gravity of diphtheritic cases depends, possibly, on the fact of this variety of bacilli, but more likely on the circumstance that other malign germs are often coincident with diphtheria. Streptococci, for instance, have been found to intensify the virulence of the diphtheritic bacilli. Besides this, there are various phases of the pathology of diphtheria which will require a correspondingly varied treatment. There may be nothing but angina, or there may be an aggravated condition of the laryngeal membrane, necessitating, according to usual practice, either tracheotomy or intubation, or there may take place such a serious affection of tonsils, or of throat and nose with membranal or suppurant obstructions, that even desperate means will be of little avail.

Each of these require an antitoxin treatment of their own, depending upon the amount of toxin in the system. This toxin increases very rapidly, and much of the therapeutic effect of antitoxin administered depends on whether the stage arrived at by the patient is of a mild form or not. Should the disease have progressed so much that the bronchi and the lungs are involved and tracheotomy itself should not seem worth the effort, then in all logic, serum, too, could do no therapeutic wonders. It is natural enough to take it for granted that if the toxin has been allowed to thrive freely without timely check, a tardy injection cannot unmake the damage already done, especially if, as is well known in cases of diphtheria, the implicit dangers are paralysis of the nerves and ganglia of the heart. Least of all can any helpful, surely not curative, effect be expected from the antitoxin treatment under conditions of mixed infection. We have already spoken of the phenomena that streptococci intensify the virulence of the diphtheria bacilli.

I may state here, that owing to the specific effect of streptococci, French physicians who formerly championed tracheotomic treatment in laryngeal stenosis, have now abandoned it and taken recourse to intubation. For it has been experienced by Roux, in the hospitals of Paris, that streptococci invade the tissues more readily when tracheotomy has been performed, that death is rarely preventable then and that infection in wards is very easy. Intubation, on the other hand, runs no such risks. It is not to be forgotten that much of the success of antitoxin treatment depends on an early administration of it. Germs are persistent fellows and the slightest brood of them will thrive even after an interval of obscuration. It is known that convalescent children will, even eight weeks after recovery, carry Klebs-Löffler bacilli when going to school. The secondary results, also, which often come in the wake of diphtheria, such as affection of the kidneys, peripheric nerves and paralysis, which not infrequently follows diphtheria, have their source in the fact that there has been a residual quantity of toxin left uncombated. This could, however, have been neutralized, if only the dose of antitoxin had been administered early enough and in sufficient quantity.

The treatment with antitoxin is about as follows, and it will be found to be surprisingly simple: the dose is proportionate to the intensity and duration of the disease. The injection is made, usually on the lateral part of the chest, but preferably on the the thigh or between the shoulder-blades. The antitoxin is absorbed within one or two hours; it produces more or less pain, which, however, disappears within twenty-four hours. There is no immediate reaction as to temperature. Sometimes a skin eruption, like urticaria, is observed, lasting for several days, occasionally for two weeks, after the injection. It is in all cases harmless. The diphtheritic membranes are usually found larger, but whiter, the next day after injection. If the larynx has not been involved before administration, it will in no case be involved after the injection of antitoxin. It is on this account that, by an early treatment with antitoxin, many cases of otherwise necessary tracheotomy are avoided.

Large doses of serum cause a critical decrease of pulse and temperature. But care must be taken that, as is often the case, these two phenomena be not traceable to complications. For it is well known that high temperature is found often in the convalescent stage of simple diphtheria. The subjective condition of the patient leaves nothing to be desired. It disposes of evident signs of distress, even in cases which are severe; and children who have become low-depressed by the drain of the disease, have been found in good humor, playful, with good appetite, a day after injection. Of course, the application of the serum has to be repeated, depending on the gravity of the case. Sometimes doses are administered the day after the first injection, regularly thereafter, or even on the same day.

There is a scale of immunizing units, with which all practitioners will do well to familiarize themselves. It is also important to have a fair estimate of the degree of toxication already present which is to be combated. Local treatment is to be applied, as usual.

Jenner gave to the world his experimental specific for prophylactic vaccination. But modern French and German scientists have given us a reliable law for inoculation for curative purposes, as well as for prophylaxis. Children may now be protected against the ravages of diphtheria. An average dose of from 100 to 200 immunizing units has been fixed for this, and reinoculation after two or three weeks is recommended.

In the period of incubation—which is a time of great concern to schools, to sanitary authorities and to public-minded citizens—the dose may be increased. Repeated application of small doses in certain intervals will secure immunity for a long period. In the New York Infant Asylum there were 130 cases treated without antitoxin with a death-rate of 26 per cent. From December 1, 1894, to February 1, 1895, fifty-nine cases were treated with antitoxin with a death-rate of 15 per cent., a decrease of 11 per cent.

From the results of the experiments of antitoxin, we can come to a safe conclusion that the administration is perfectly harmless and it positively possesses prophylactic and curative properties, if applied in proper quantity. I think the trouble is that too small a quantity is given and there must be enough antitoxin administered to neutralize the toxin in the system. It is evident that it requires a larger dose of antitoxin to cure a case of several days' standing than to check an infection in its incipiency.

1. Read before the Detroit Academy of Medicine, Detroit, Mich., March 11, 1895.

2. See, for an exceptionally clear account of the successive steps which led to the formulation of Behring's law, *Krieger's Serum Therapy*, p. 11.

535 Woodward Avenue.

---

The Income Tax.—At the request of the chairman of the board of censors, Mr. Taylor, the counsel for the Philadelphia County Medical Society, sent a written opinion concerning the income tax in relation to physicians. A physician's office, or that part of his dwelling used as an office and not the whole house, was exempt in estimating his income.—*Times and Register, April 20, 1895.*

